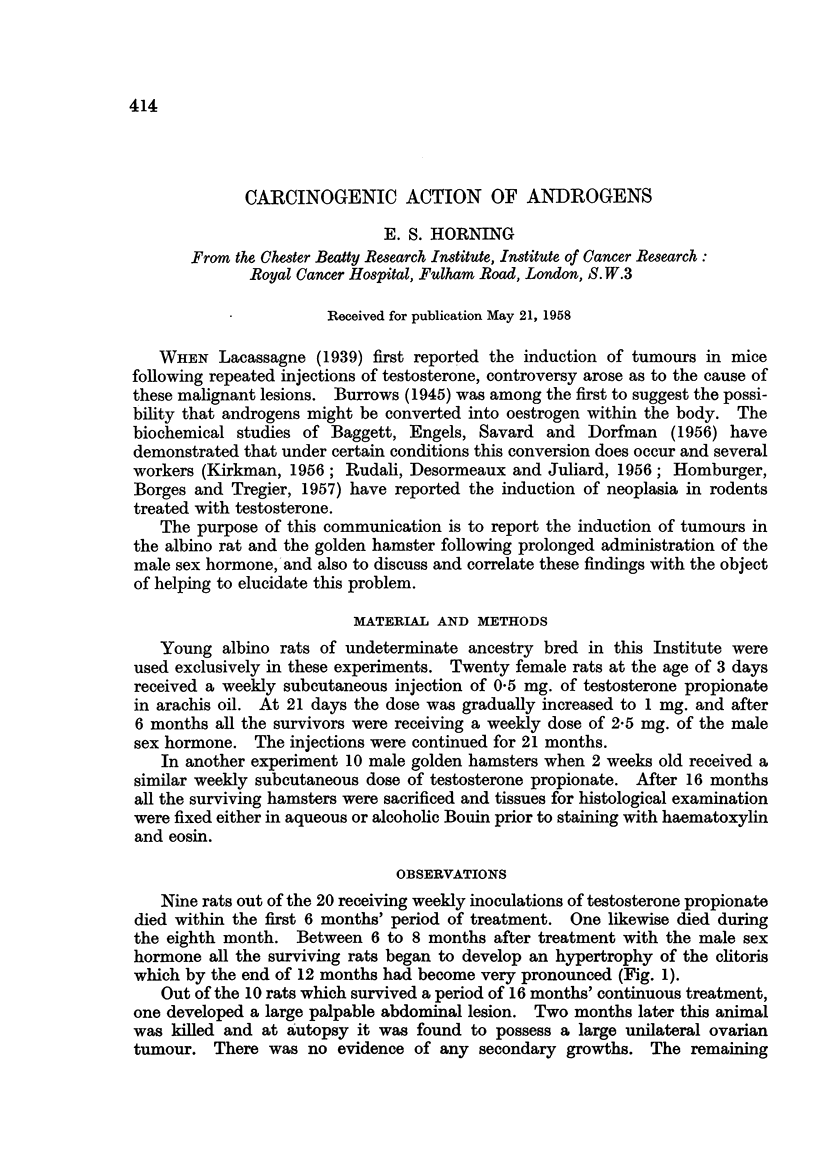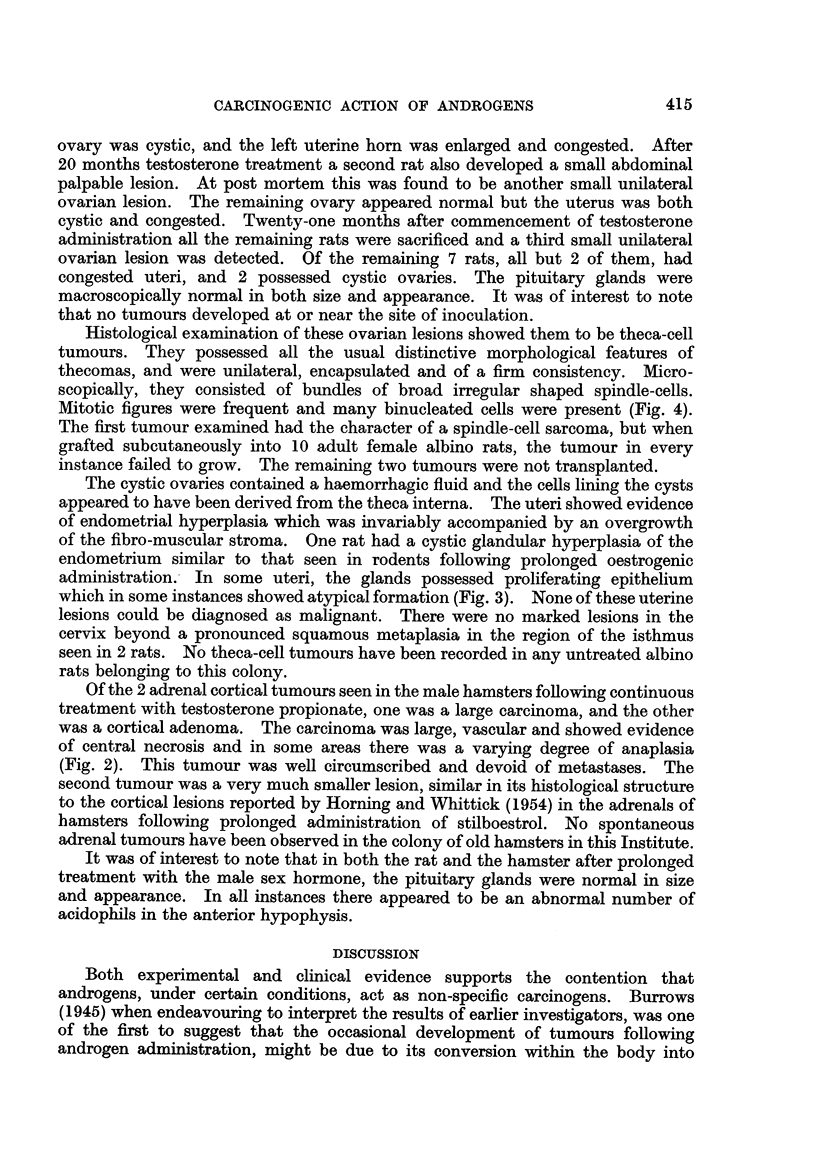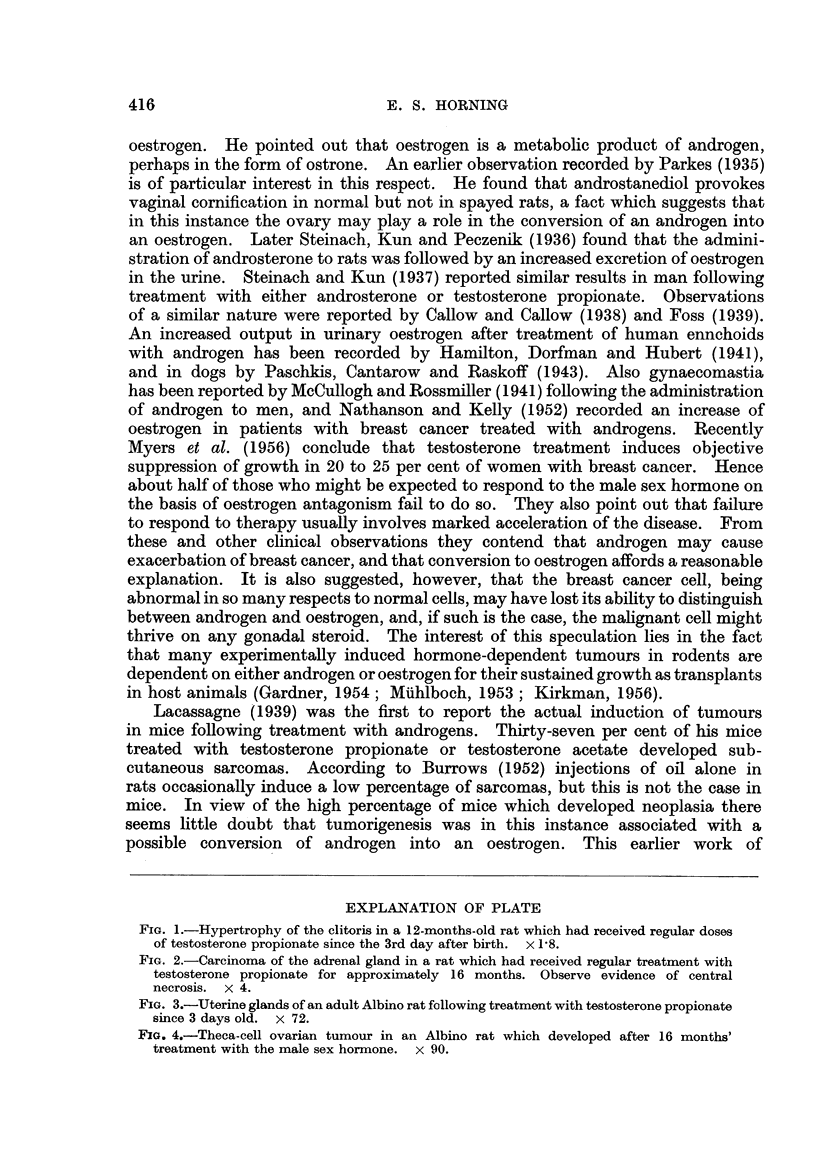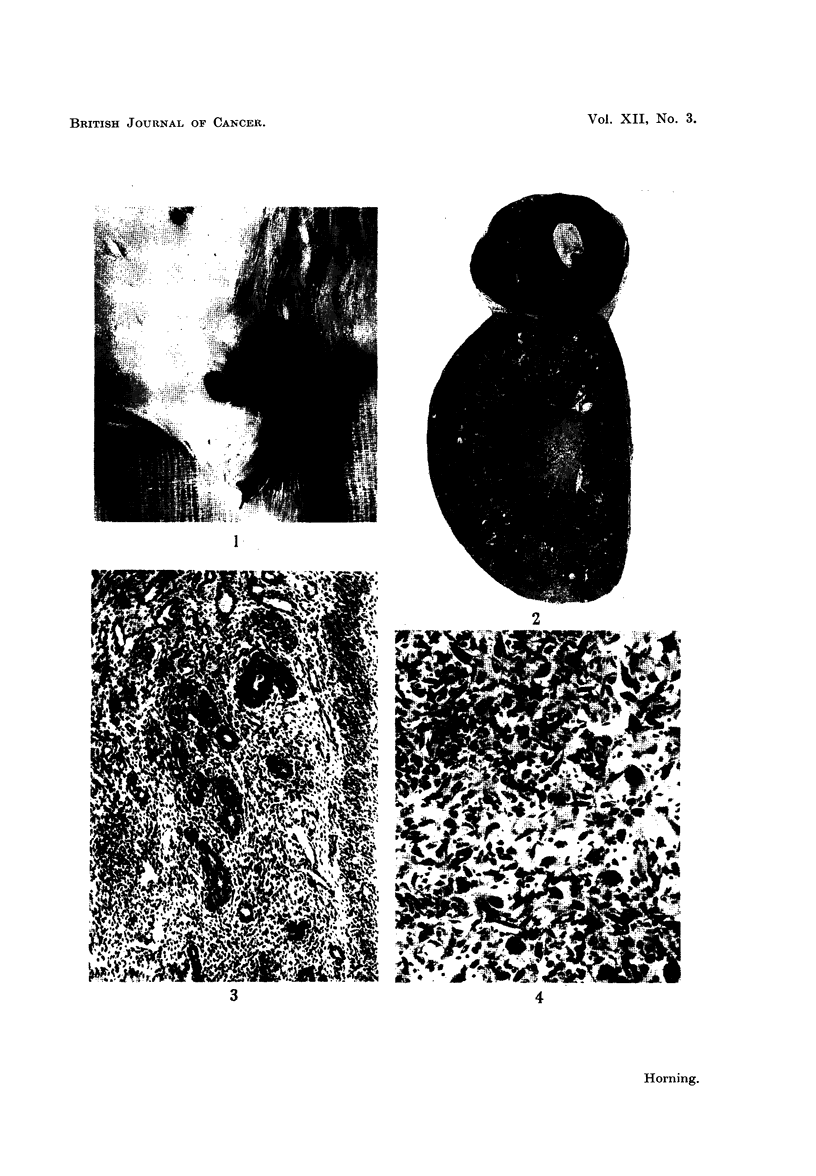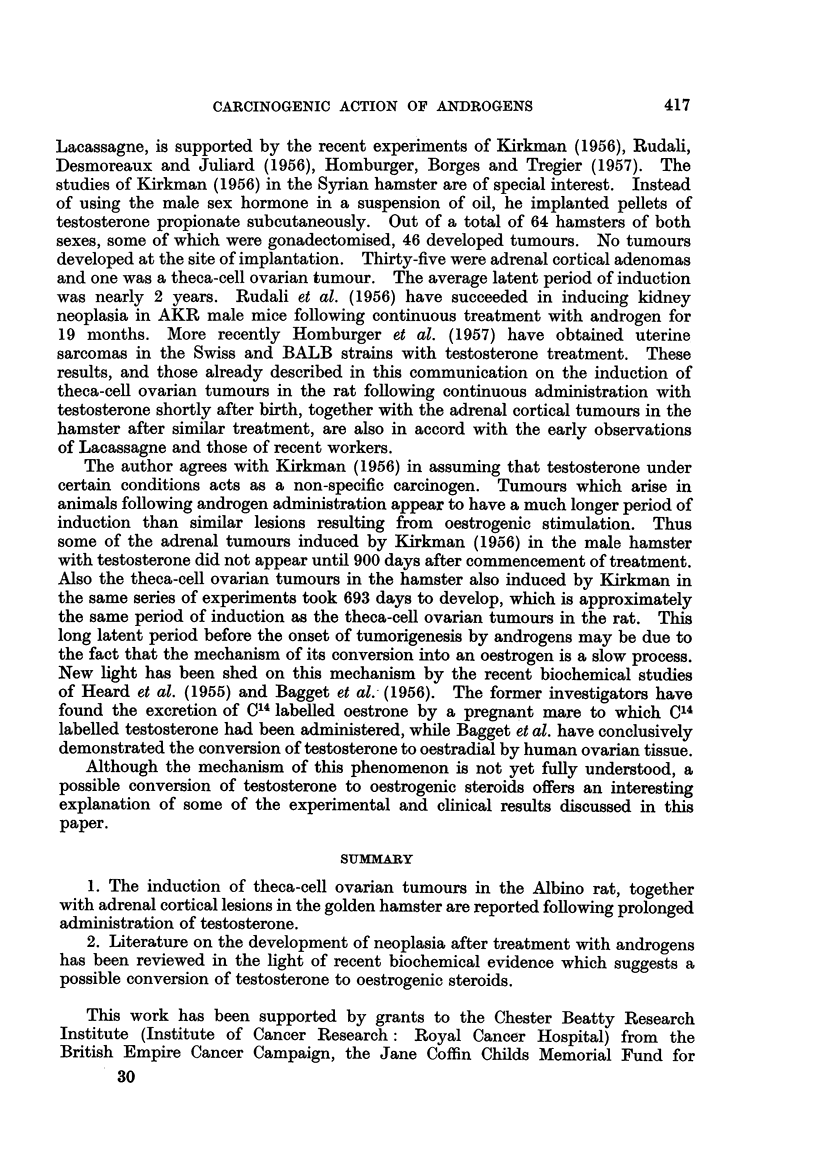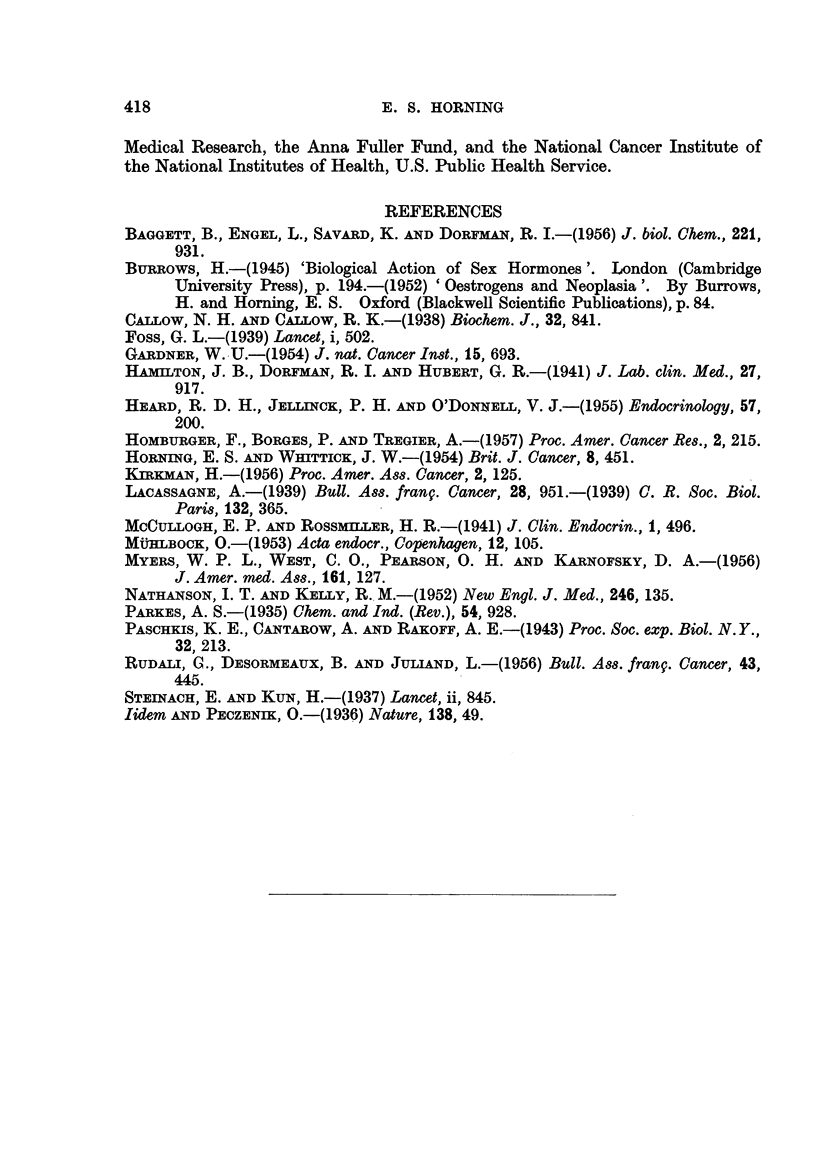# Carcinogenic Action of Androgens

**DOI:** 10.1038/bjc.1958.49

**Published:** 1958-09

**Authors:** E. S. Horning

## Abstract

**Images:**


### CARCINOGENIC ACTION OF ANDROGENS

E. S. HORNING

*From the Chester Beatty Research Institute, Institute of Cancer Research : Royal Cancer Hospital, Fulham Road, London, S.W.3*

Received for publication May 21, 1958

WHEN Lacassagne (1939) first reported the induction of tumours in mice following repeated injections of testosterone, controversy arose as to the cause of these malignant lesions. Burrows (1945) was among the first to suggest the possibility that androgens might be converted into oestrogen within the body. The biochemical studies of Baggett, Engels, Savard and Dorfman (1956) have demonstrated that under certain conditions this conversion does occur and several workers (Kirkman, 1956 ; Rudali, Desormeaux and Juliard, 1956 ; Homburger, Borges and Tregier, 1957) have reported the induction of neoplasia in rodents treated with testosterone.

The purpose of this communication is to report the induction of tumours in the albino rat and the golden hamster following prolonged administration of the male sex hormone, and also to discuss and correlate these findings with the object of helping to elucidate this problem.

### MATERIAL AND METHODS

Young albino rats of undeterminate ancestry bred in this Institute were used exclusively in these experiments. Twenty female rats at the age of 3 days received a weekly subcutaneous injection of 0·5 mg. of testosterone propionate in arachis oil. At 21 days the dose was gradually increased to 1 mg. and after 6 months all the survivors were receiving a weekly dose of 2·5 mg. of the male sex hormone. The injections were continued for 21 months.

In another experiment 10 male golden hamsters when 2 weeks old received a similar weekly subcutaneous dose of testosterone propionate. After 16 months all the surviving hamsters were sacrificed and tissues for histological examination were fixed either in aqueous or alcoholic Bouin prior to staining with haematoxylin and eosin.

### OBSERVATIONS

Nine rats out of the 20 receiving weekly inoculations of testosterone propionate died within the first 6 months' period of treatment. One likewise died during the eighth month. Between 6 to 8 months after treatment with the male sex hormone all the surviving rats began to develop an hypertrophy of the clitoris which by the end of 12 months had become very pronounced (Fig. 1).

Out of the 10 rats which survived a period of 16 months' continuous treatment, one developed a large palpable abdominal lesion. Two months later this animal was killed and at autopsy it was found to possess a large unilateral ovarian tumour. There was no evidence of any secondary growths. The remaining ovary was cystic, and the left uterine horn was enlarged and congested. After 20 months testosterone treatment a second rat also developed a small abdominal palpable lesion. At post mortem this was found to be another small unilateral ovarian lesion. The remaining ovary appeared normal but the uterus was both cystic and congested. Twenty-one months after commencement of testosterone administration all the remaining rats were sacrificed and a third small unilateral ovarian lesion was detected. Of the remaining 7 rats, all but 2 of them, had congested uteri, and 2 possessed cystic ovaries. The pituitary glands were macroscopically normal in both size and appearance. It was of interest to note that no tumours developed at or near the site of inoculation.

Histological examination of these ovarian lesions showed them to be theca-cell tumours. They possessed all the usual distinctive morphological features of thecomas, and were unilateral, encapsulated and of a firm consistency. Microscopically, they consisted of bundles of broad irregular shaped spindle-cells. Mitotic figures were frequent and many binucleated cells were present (Fig. 4). The first tumour examined had the character of a spindle-cell sarcoma, but when grafted subcutaneously into 10 adult female albino rats, the tumour in every instance failed to grow. The remaining two tumours were not transplanted.

The cystic ovaries contained a haemorrhagic fluid and the cells lining the cysts appeared to have been derived from the theca interna. The uteri showed evidence of endometrial hyperplasia which was invariably accompanied by an overgrowth of the fibro-muscular stroma. One rat had a cystic glandular hyperplasia of the endometrium similar to that seen in rodents following prolonged oestrogenic administration. In some uteri, the glands possessed proliferating epithelium which in some instances showed atypical formation (Fig. 3). None of these uterine lesions could be diagnosed as malignant. There were no marked lesions in the cervix beyond a pronounced squamous metaplasia in the region of the isthmus seen in 2 rats. No theca-cell tumours have been recorded in any untreated albino rats belonging to this colony.

Of the 2 adrenal cortical tumours seen in the male hamsters following continuous treatment with testosterone propionate, one was a large carcinoma, and the other was a cortical adenoma. The carcinoma was large, vascular and showed evidence of central necrosis and in some areas there was a varying degree of anaplasia (Fig. 2). This tumour was well circumscribed and devoid of metastases. The second tumour was a very much smaller lesion, similar in its histological structure to the cortical lesions reported by Horning and Whittick (1954) in the adrenals of hamsters following prolonged administration of stilboestrol. No spontaneous adrenal tumours have been observed in the colony of old hamsters in this Institute.

It was of interest to note that in both the rat and the hamster after prolonged treatment with the male sex hormone, the pituitary glands were normal in size and appearance. In all instances there appeared to be an abnormal number of acidophils in the anterior hypophysis.

### DISCUSSION

Both experimental and clinical evidence supports the contention that androgens, under certain conditions, act as non-specific carcinogens. Burrows (1945) when endeavouring to interpret the results of earlier investigators, was one of the first to suggest that the occasional development of tumours following androgen administration, might be due to its conversion within the body into oestrogen. He pointed out that oestrogen is a metabolic product of androgen, perhaps in the form of ostrone. An earlier observation recorded by Parkes (1935) is of particular interest in this respect. He found that androstanediol provokes vaginal cornification in normal but not in spayed rats, a fact which suggests that in this instance the ovary may play a role in the conversion of an androgen into an oestrogen. Later Steinach, Kun and Peczenik (1936) found that the administration of androsterone to rats was followed by an increased excretion of oestrogen in the urine. Steinach and Kun (1937) reported similar results in man following treatment with either androsterone or testosterone propionate. Observations of a similar nature were reported by Callow and Callow (1938) and Foss (1939). An increased output in urinary oestrogen after treatment of human ennchoids with androgen has been recorded by Hamilton, Dorfman and Hubert (1941), and in dogs by Paschkis, Cantarow and Raskoff (1943). Also gynaecomastia has been reported by McCullogh and Rossmiller (1941) following the administration of androgen to men, and Nathanson and Kelly (1952) recorded an increase of oestrogen in patients with breast cancer treated with androgens. Recently Myers *et al.* (1956) conclude that testosterone treatment induces objective suppression of growth in 20 to 25 per cent of women with breast cancer. Hence about half of those who might be expected to respond to the male sex hormone on the basis of oestrogen antagonism fail to do so. They also point out that failure to respond to therapy usually involves marked acceleration of the disease. From these and other clinical observations they contend that androgen may cause exacerbation of breast cancer, and that conversion to oestrogen affords a reasonable explanation. It is also suggested, however, that the breast cancer cell, being abnormal in so many respects to normal cells, may have lost its ability to distinguish between androgen and oestrogen, and, if such is the case, the malignant cell might thrive on any gonadal steroid. The interest of this speculation lies in the fact that many experimentally induced hormone-dependent tumours in rodents are dependent on either androgen or oestrogen for their sustained growth as transplants in host animals (Gardner, 1954 ; Mühlboch, 1953 ; Kirkman, 1956).

Lacassagne (1939) was the first to report the actual induction of tumours in mice following treatment with androgens. Thirty-seven per cent of his mice treated with testosterone propionate or testosterone acetate developed subcutaneous sarcomas. According to Burrows (1952) injections of oil alone in rats occasionally induce a low percentage of sarcomas, but this is not the case in mice. In view of the high percentage of mice which developed neoplasia there seems little doubt that tumorigenesis was in this instance associated with a possible conversion of androgen into an oestrogen. This earlier work of

---

EXPLANATION OF PLATE

FIG. 1.—Hypertrophy of the clitoris in a 12-months-old rat which had received regular doses of testosterone propionate since the 3rd day after birth. × 1·8.

FIG. 2.—Carcinoma of the adrenal gland in a rat which had received regular treatment with testosterone propionate for approximately 16 months. Observe evidence of central necrosis. × 4.

FIG. 3.—Uterine glands of an adult Albino rat following treatment with testosterone propionate since 3 days old. × 72.

FIG. 4.—Theca-cell ovarian tumour in an Albino rat which developed after 16 months' treatment with the male sex hormone. × 90.

1

2

3

4

Horning.

Lacassagne, is supported by the recent experiments of Kirkman (1956), Rudali, Desmoreaux and Juliard (1956), Homburger, Borges and Tregier (1957). The studies of Kirkman (1956) in the Syrian hamster are of special interest. Instead of using the male sex hormone in a suspension of oil, he implanted pellets of testosterone propionate subcutaneously. Out of a total of 64 hamsters of both sexes, some of which were gonadectomised, 46 developed tumours. No tumours developed at the site of implantation. Thirty-five were adrenal cortical adenomas and one was a theca-cell ovarian tumour. The average latent period of induction was nearly 2 years. Rudali *et al.* (1956) have succeeded in inducing kidney neoplasia in AKR male mice following continuous treatment with androgen for 19 months. More recently Homburger *et al.* (1957) have obtained uterine sarcomas in the Swiss and BALB strains with testosterone treatment. These results, and those already described in this communication on the induction of theca-cell ovarian tumours in the rat following continuous administration with testosterone shortly after birth, together with the adrenal cortical tumours in the hamster after similar treatment, are also in accord with the early observations of Lacassagne and those of recent workers.

The author agrees with Kirkman (1956) in assuming that testosterone under certain conditions acts as a non-specific carcinogen. Tumours which arise in animals following androgen administration appear to have a much longer period of induction than similar lesions resulting from oestrogenic stimulation. Thus some of the adrenal tumours induced by Kirkman (1956) in the male hamster with testosterone did not appear until 900 days after commencement of treatment. Also the theca-cell ovarian tumours in the hamster also induced by Kirkman in the same series of experiments took 693 days to develop, which is approximately the same period of induction as the theca-cell ovarian tumours in the rat. This long latent period before the onset of tumorigenesis by androgens may be due to the fact that the mechanism of its conversion into an oestrogen is a slow process. New light has been shed on this mechanism by the recent biochemical studies of Heard *et al.* (1955) and Bagget *et al.* (1956). The former investigators have found the excretion of $C^{14}$ labelled oestrone by a pregnant mare to which $C^{14}$ labelled testosterone had been administered, while Bagget *et al.* have conclusively demonstrated the conversion of testosterone to oestradial by human ovarian tissue.

Although the mechanism of this phenomenon is not yet fully understood, a possible conversion of testosterone to oestrogenic steroids offers an interesting explanation of some of the experimental and clinical results discussed in this paper.

### SUMMARY

1. The induction of theca-cell ovarian tumours in the Albino rat, together with adrenal cortical lesions in the golden hamster are reported following prolonged administration of testosterone.

2. Literature on the development of neoplasia after treatment with androgens has been reviewed in the light of recent biochemical evidence which suggests a possible conversion of testosterone to oestrogenic steroids.

This work has been supported by grants to the Chester Beatty Research Institute (Institute of Cancer Research: Royal Cancer Hospital) from the British Empire Cancer Campaign, the Jane Coffin Childs Memorial Fund for

Medical Research, the Anna Fuller Fund, and the National Cancer Institute of the National Institutes of Health, U.S. Public Health Service.

### REFERENCES

BAGGETT, B., ENGEL, L., SAVARD, K. AND DORFMAN, R. I.—(1956) *J. biol. Chem.*, **221**, 931.

BURROWS, H.—(1945) 'Biological Action of Sex Hormones'. London (Cambridge University Press), p. 194.—(1952) 'Oestrogens and Neoplasia'. By Burrows, H. and Horning, E. S. Oxford (Blackwell Scientific Publications), p. 84.

CALLOW, N. H. AND CALLOW, R. K.—(1938) *Biochem. J.*, **32**, 841.

FOSS, G. L.—(1939) *Lancet*, i, 502.

GARDNER, W. U.—(1954) *J. nat. Cancer Inst.*, **15**, 693.

HAMILTON, J. B., DORFMAN, R. I. AND HUBERT, G. R.—(1941) *J. Lab. clin. Med.*, **27**, 917.

HEARD, R. D. H., JELLINCK, P. H. AND O'DONNELL, V. J.—(1955) *Endocrinology*, **57**, 200.

HOMBURGER, F., BORGES, P. AND TREGIER, A.—(1957) *Proc. Amer. Cancer Res.*, **2**, 215.

HORNING, E. S. AND WHITTICK, J. W.—(1954) *Brit. J. Cancer*, **8**, 451.

KIRKMAN, H.—(1956) *Proc. Amer. Ass. Cancer*, **2**, 125.

LACASSAGNE, A.—(1939) *Bull. Ass. franç. Cancer*, **28**, 951.—(1939) *C. R. Soc. Biol. Paris*, **132**, 365.

MCCULLOGH, E. P. AND ROSSMILLER, H. R.—(1941) *J. Clin. Endocrin.*, **1**, 496.

MÜHLBOCK, O.—(1953) *Acta endocr., Copenhagen*, **12**, 105.

MYERS, W. P. L., WEST, C. O., PEARSON, O. H. AND KARNOFSKY, D. A.—(1956) *J. Amer. med. Ass.*, **161**, 127.

NATHANSON, I. T. AND KELLY, R. M.—(1952) *New Engl. J. Med.*, **246**, 135.

PARKES, A. S.—(1935) *Chem. and Ind.* (*Rev.*), **54**, 928.

PASCHKIS, K. E., CANTAROW, A. AND RAKOFF, A. E.—(1943) *Proc. Soc. exp. Biol. N.Y.*, **32**, 213.

RUDALI, G., DESORMEAUX, B. AND JULIAND, L.—(1956) *Bull. Ass. franç. Cancer*, **43**, 445.

STEINACH, E. AND KUN, H.—(1937) *Lancet*, ii, 845.

*Iidem* AND PECZENIK, O.—(1936) *Nature*, **138**, 49.